# The Relationship Between Polypharmacy and Hospital-stay Duration: A Retrospective Study

**DOI:** 10.7759/cureus.7267

**Published:** 2020-03-14

**Authors:** Nobuhiko Fukuba, Mutsuko Nishida, Miki Hayashi, Natsuko Furukawa, Hitomi Ishitobi, Makoto Nagaoka, Yoshiko Takahashi, Hiroyuki Fukuhara, Mika Yuki, Yoshinori Komazawa, Shuichi Sato, Toshihiro Shizuku

**Affiliations:** 1 Internal Medicine, Izumo-City General Medical Center, Izumo, JPN; 2 Pharmacy, Izumo-City General Medical Center, Izumo, JPN

**Keywords:** hospital stay period, infection, polypharmacy

## Abstract

Background

There have been very few studies on the association of polypharmacy with clinical course. In this paper, we seek to evaluate the relationship between polypharmacy and hospitalization period.

Methods

We retrospectively analyzed 322 patients hospitalized from February to September 2017, after excluding short-term and orthopedic cases. Patients with polypharmacy were defined as those who were prescribed more than five drugs at the time of admission. The primary endpoint for all subjects regardless of polypharmacy was the hospitalization period. Using Mann-Whitney U test results, we compared the average number of hospital days between patients with and without polypharmacy. Secondary endpoints were hospitalization period with and without polypharmacy for each disease type.

Results

The hospitalization period was significantly extended for patients with polypharmacy as compared to those without (31.6 vs. 23.2 days, p: 0.002). Those with an infection had significantly longer hospitalization than those without polypharmacy (27.6 vs. 18.1 days, p: 0.007). Malignancy, heart disease, and cerebrovascular disease did not have a significant effect on hospitalization regardless of polypharmacy.

Conclusion

Polypharmacy is related to an extended hospitalization period and is found to occur more frequently in patients hospitalized for an infection.

## Introduction

Polypharmacy refers to multiple drug administration and is considered to be a factor related to adverse drug events relative to the number of medications prescribed [1]. Notably, elderly individuals show changes in pharmacokinetics and pharmacodynamics due to aging, and the use of multiple drugs can lead to adverse events [2]. However, the potential harmful effects of polypharmacy can vary depending on types of medicine and disease conditions, though few studies have been conducted to examine the association of polypharmacy with clinical course in individual patients. In the present study, we examined cases with an extended stay at our hospital and evaluated differences according to the number of medications administered.

## Materials and methods

Study design

This retrospective study was conducted at Izumo-City General Medical Center in Japan. The primary endpoint was hospitalization period of patients with or without polypharmacy, while the secondary endpoints were hospitalization period with and without polypharmacy for each disease type.

Subjects

We retrospectively analyzed the records of 846 patients hospitalized at our hospital from February to September 2017. After excluding short-term and orthopedic hospitalization cases, 322 were enrolled.

Methods

We noted the age, gender, primary disease leading to hospitalization, number and therapeutic category of medications administered, and period of hospital stay of the enrolled patients. Polypharmacy was defined as the administration of more than five different drugs at the time of admission. The average number of hospital days was compared between those with and without polypharmacy using Mann-Whitney U test findings. We calculated the correlation coefficient between the number of medications and the length of hospitalization for each patient. Furthermore, conditions that led to hospitalization were classified into five groups; infection, malignant disease, heart disease, cerebrovascular disease, and others. Using Mann-Whitney U test findings, the average number of hospital days for each group was compared between the polypharmacy and non-polypharmacy groups. The IBM SPSS Statistics software Version 19 (IBM Corporation, Armonk, NY) was used for all statistical analyses, and a p-value of <0.05 was considered to be statistically significant.

## Results

**Figure 1:**
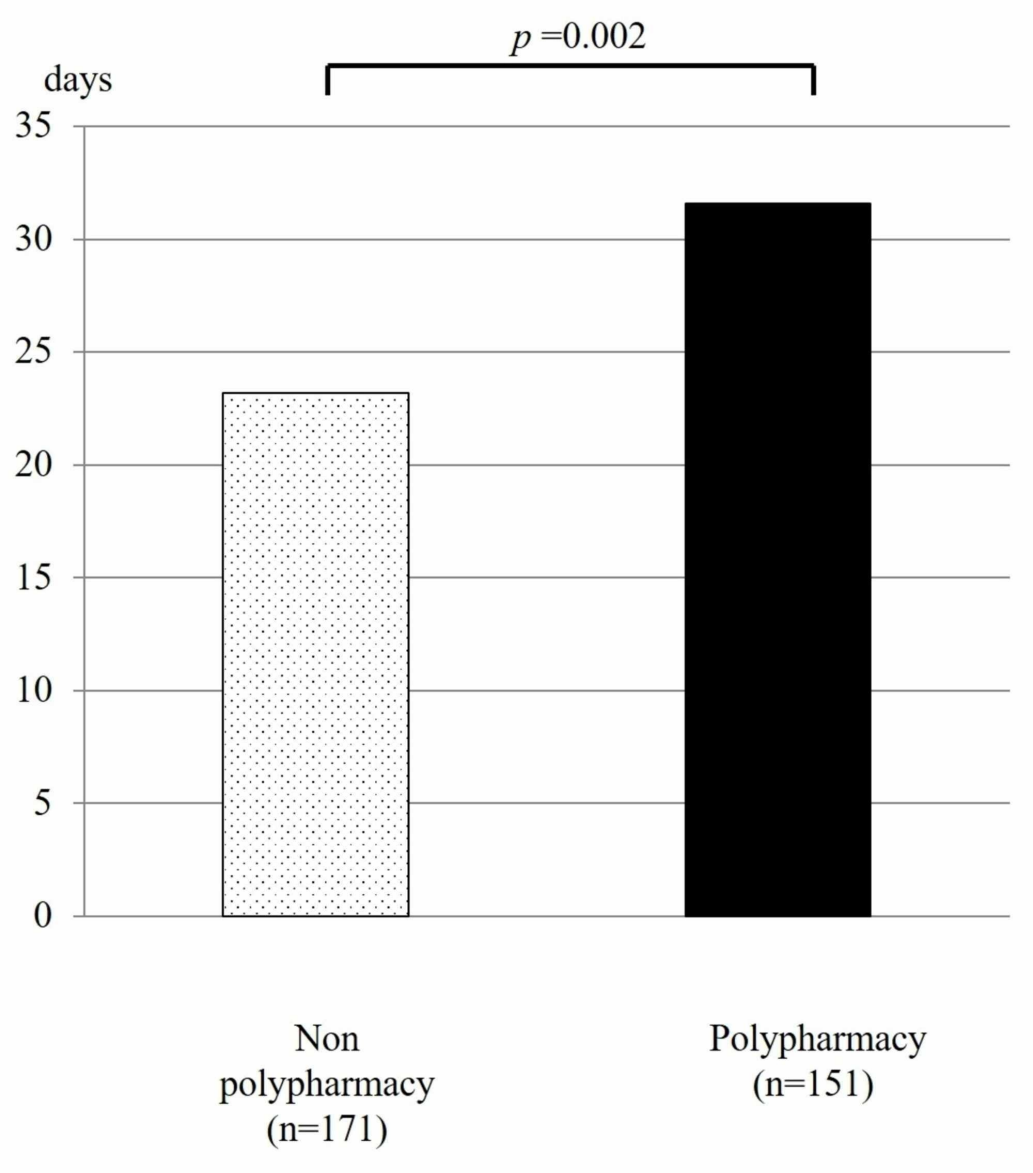
The hospitalization period of patients with or without polypharmacy

Of the 846 patients hospitalized during the study period, 322 were enrolled, after excluding those with a short-term or orthopedic hospitalization. The average age of the enrolled patients was 80.4 years and the average number of medications being administered at the time of admission was 5.4, with 151 (47%) cases categorized as polypharmacy and 171 (53%) as non-polypharmacy. There was no significant difference in regard to the background characteristics of the polypharmacy and non-polypharmacy groups, though the hospitalization period was significantly longer for patients with polypharmacy (mean ±SD: 31.6 ±2.8 vs. 23.2 ±2.2 days, p: 0.002) (Figure 1).

**Figure 2:**
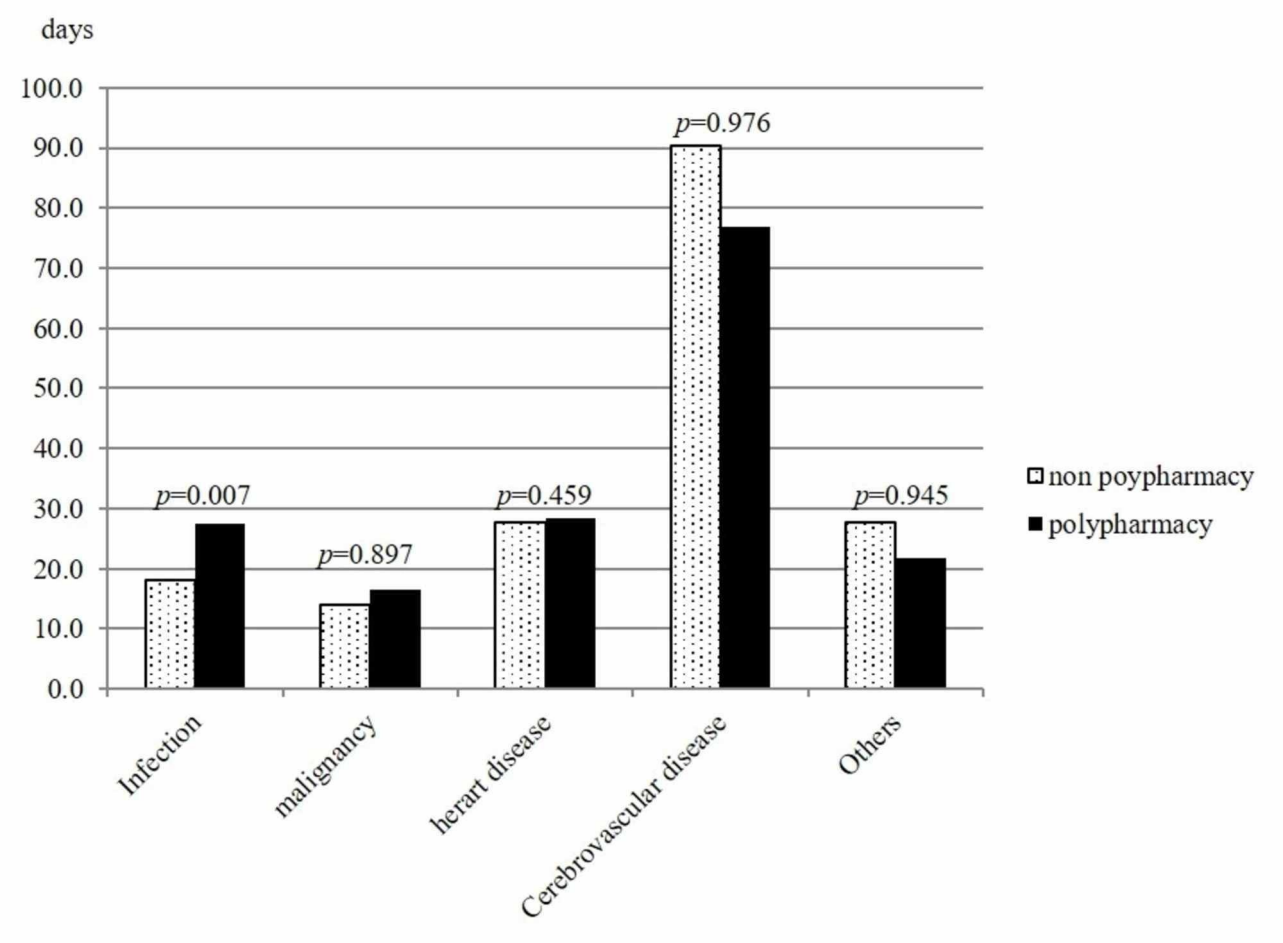
Mean hospitalization period (days) for patients with or without polypharmacy by disease group

Next, we divided the 322 subjects into disease groups and examined the relationship of polypharmacy with the extended length of stay in each. The number of patients in the infection, malignant disease, cerebrovascular disease, heart disease, and others groups was 166 (52%), 84 (26%), 32 (10%), 26 (8.1%), and 14 (4.3%), respectively. In the infection group, the hospitalization period of patients with polypharmacy was significantly longer as compared to those without polypharmacy (mean ±SD: 27.6 ±3.1 vs. 18.1 ±1.5days, p =0.007), while there was no significant difference between with and without polypharmacy in the other 4 disease groups (Figure 2).

## Discussion

Polypharmacy is a condition in which several medications, often more than clinically required, are prescribed, with the classification generally applied when the number of drugs administered is five or more. It has been reported that adverse drug events increase when the number of concomitant drugs exceeds 5 [1]. In the present polypharmacy group, comprised of patients administered more than five different drugs, the hospitalization period was significantly longer as compared to the non-polypharmacy group. Multiple drug administration is suspected to cause adverse events that lengthen hospitalization. For example, one of the most serious adverse events encountered by hospitalized patients is cognitive decline, which is thought to be related to benzodiazepine and/or antihistamine administration. While adverse events are rarely encountered in cases with a single agent, metabolism can be inhibited when more medications are added, resulting in an increase in blood concentration when given in combination with other agents.

Another possible reason for the long hospital stay in the present polypharmacy group is that those patients typically had multiple underlying diseases. There may be fewer treatment options for a particular condition if several underlying conditions are also present. Because of the presence of underlying disease, optimal treatment is not available and the therapy period may become prolonged. Alternatively, an underlying disease may be exacerbated by the primary condition, thus requiring specific treatment for the underlying complication.

In the present study, the number of underlying diseases in our patients was a confounding factor, as drug number increased in cases with several underlying diseases present, leading to polypharmacy. Polypharmacy is a complex problem and a large variety of factors, such as type and dose of each drug, type and degree of underlying disease, patient age, patient organ function status, and those in combinations must be considered. It is clear that polypharmacy is an important issue to be addressed, with additional investigations needed regarding drugs with significant influence and which patients are most likely affected.

When the analysis was performed based on disease group, only polypharmacy patients with an infectious disease had their hospitalization period extended. As for the reasons for that finding, it was noted that approximately half of the infectious cases in this study were pneumonia. Although results demonstrating that polypharmacy itself depresses swallowing have been presented, some medications routinely administered to elderly polypharmacy patients have been reported to induce aspiration pneumonia. Drugs with anticholinergic activity are likely to cause xerostomia, delay in food transport in the oral cavity and pharynx, and decreased swallowing function [3]. These drugs include muscarinic receptor antagonists such as overactive bladder therapeutics, antihistamines, and tricyclic antidepressants. On the other hand, proton pump inhibitor administration is also considered to increase the risk of pneumonia. In a case-control study of approximately 40,000 patients in Denmark, the incidence of pneumonia was found to be significantly higher in those receiving proton pump inhibitors (odds ratio: 1.5) [4]. Polypharmacy patients are more likely to be given such high-risk drugs than non-polypharmacy patients. In the present infectious disease group, the percentage of patients taking at least one of these high-risk drugs was greater in the polypharmacy group (76.2% vs. 34.1%). Therefore, the polypharmacy group likely included a number of patients at high risk for pneumonia and the resolution of their condition was prolonged, resulting in an extended hospitalization period. In contrast to infectious diseases, cardiovascular, cerebrovascular, and malignant diseases are rarely caused by drugs, and polypharmacy did not extend the hospitalization period in any of those groups in our study. It is unlikely that polypharmacy for all diseases extends the hospitalization period and its influence differs depending on the primarily targeted disease. Nevertheless, the hospitalization period for infectious diseases such as pneumonia is affected by polypharmacy.

## Conclusions

We can conclude that polypharmacy is related to an extended period of hospitalization, especially in patients hospitalized for infectious diseases. Polypharmacy is an important issue to be addressed, and further investigations are required to analyze its effects in a comprehensive manner.
